# Volvulus of the ileal pouch–anal anastomosis: a meta-narrative systematic review of frequency, diagnosis, and treatment outcomes

**DOI:** 10.1093/gastro/goz045

**Published:** 2019-09-17

**Authors:** Muhammad Jawoosh, Samir Haffar, Parakkal Deepak, Alyssa Meyers, Amy L Lightner, David W Larson, Laura H Raffals, M Hassan Murad, Navtej Buttar, Fateh Bazerbachi

**Affiliations:** 1 Division of Gastroenterology and Hepatology, Dessau Municipal Hospital, Auenweg 38, Dessau-Rosslau, Germany; 2 Digestive Center for Diagnosis and Treatment, Damascus, Syrian Arab Republic; 3 Division of Gastroenterology, John T. Milliken Department of Medicine, Washington University School of Medicine, St. Louis, MO, USA; 4 Division of Gastroenterology and Hepatology, Mayo Clinic, Rochester, MN, USA; 5 Department of Colorectal Surgery, Cleveland Clinic, Cleveland, OH, USA; 6 Division of Colon and Rectal Surgery, Mayo Clinic, Rochester, MN, USA; 7 Robert D and Patricia E Kern Center for the Science of Health Care Delivery, Mayo Clinic, Rochester, MN, USA; 8 Division of Gastroenterology, Massachusetts General Hospital, Boston, MA, USA

**Keywords:** familial adenomatous polyposis, ulcerative colitis, ileal pouch–anal anastomosis, pouch volvulus, systematic review

## Abstract

**Background:**

Proctocolectomy with ileal pouch–anal anastomosis (IPAA) is the surgical procedure of choice for medically refractory ulcerative colitis and familial adenomatous polyposis. While rare, a pouch volvulus can occur. We aimed to determine the frequency, presentation, and management approach of pouch volvulus in patients with IPAA.

**Methods:**

A systematic search of published literature was performed by a medical reference librarian on 10 August 2018 and two independent reviewers identified relevant publications, extracted data, and assessed the methodological quality based on a validated tool. A retrospective review of the Mayo Clinic electronic medical records identified one case of pouch volvulus between January 2008 and August 2018.

**Results:**

The frequency of pouch volvulus from one large published study reporting long-term outcomes of IPAA was 0.18% (3/1,700). A total of 22 patients (18 ulcerative colitis) were included (median age 32 years, 73% females). Median time to volvulus after IPAA was 36 months while median interval to volvulus diagnosis from symptom onset was 24 hours. Abdominal pain was the most commonly reported symptom (76%). The diagnosis was made primarily by abdominal computed tomography (13/17 patients, 76%). Endoscopic treatment was successful in 1 of 11 patients (9%). Surgery was performed in 20 patients and pouch-pexy and pouch excision were the most frequent surgical operations. A redo IPAA was performed in five patients (25%).

**Conclusion:**

Pouch volvulus is a rare but serious complication of IPAA and should be suspected even in the absence of obstruction symptoms. Endoscopic treatment often fails and surgery is effective when performed early.

## Introduction

Proctocolectomy with ileal pouch–anal anastomosis (IPAA) is the surgical procedure of choice for the treatment of medically refractory ulcerative colitis (UC) and familial adenomatous polyposis (FAP). Since its first description in 1978 by Parks and Nicholls, the procedure evolved to include the creation of three types of pouch configurations (J, S, and W). The overall morbidity of pouch construction is reported at between 8% and 28%, with a low mortality rate between 0% and 4% [1]. With increased experience among surgeons in the four decades since its conception, pouch complications have significantly decreased [2]. The less technically demanding J-pouch is generally preferred by surgeons [3].

Pouch volvulus is a rare mechanical complication of IPAA surgery and little is known about its frequency, natural history, and optimal treatment algorithm. This unique complication may portend sinister outcomes such as pouch necrosis and ischemia, leading to pouch excision and permanent stoma. Prompt and early diagnosis of pouch volvulus is critical and increased clinical awareness of this entity is important. In this systematic review and pooled analysis, we aim to delineate the frequency, clinical presentation, diagnostic approach, management, and outcomes of IPAA volvulus.

## Methods

This systematic review is reported according to the preferred reporting items for systematic reviews and meta-analyses (PRISMA) with an a priori study protocol [4].

### Data sources and search strategies

A medical reference librarian conducted a comprehensive, language-unrestricted search of several databases from each database's inception to 10 August 2018. The data sources and search strategies are provided in Supplementary File 1. We also searched the first 300 entries of Google Scholar using the terms ‘ileal pouch–anal anastomosis’ to identify unpublished cases. Reference lists of relevant publications were manually reviewed for additional publications. Using Mayo Clinic’s ‘Advanced cohort explorer’ clinical database, we performed a search for consecutive patients from 1 January 2008 to 10 August 2018, who were treated at the Mayo Clinic in Rochester, MN with a diagnosis of pouch volvulus.

### Study parameters based on the following definitions

#### The frequency of pouch volvulus

Given the rarity of pouch volvulus, the estimation of its frequency was based on published studies including at least 1,000 patients with IPAA and reporting the long-term outcome. In the case of multiple publications emanating from the same center at different periods of time, we elected to include the most recent or the most relevant publication to estimate the frequency.

#### Inclusion criteria

We included case reports and case series that reported the diagnosis of pouch volvulus in patients with IPAA, affirming this diagnosis by different imaging modalities, endoscopy, or surgery with sufficient data to be reported individually.

#### Delay in the diagnosis of pouch volvulus

We considered that there was a delay in diagnosis of pouch volvulus when the diagnosis was made more than 12 hours after the onset of symptoms because the intestine can compensate for approximately 75% reduction of its mesenteric blood flow during this period without substantial injury [5].

#### Exclusion criteria

We excluded duplicated studies and studies with insufficient clinical data. In addition, we excluded cases of small-bowel volvulus without actual pouch volvulus in patients with IPAA and cases of pouch twist encountered during the surgical intervention [6].

### Data extraction

Two independent reviewers (M.J. and S.H.) evaluated the studies based on the selection criteria and extracted the relevant data onto a standardized form. The data included year of publication, country of origin, publication language, publication format (full-text article, letter to the editors, abstract form), type of study (case report, case series), age, gender, ethnicity, medical history, body mass index (BMI), indication of IPAA, type of pouch (J, S, W), IPAA creation-to-volvulus interval, clinical symptoms at presentation (abdominal pain, nausea and vomiting, abdominal distension, failure to pass feces and flatus, pyrexia), serological exams, radiological exams (plain abdominal radiography, Gastrografin enema, abdominal computed tomography), endoscopic findings, endoscopic and surgical treatment, axis of volvulus, complications of volvulus, post-surgical complications, duration of follow-up after therapy, recurrence of volvulus, and the final outcome. Disagreements between the reviewers were settled by discussion and adjudication by the corresponding author (F.B.).

### Assessment of methodological quality of included studies

The quality of the included reports was determined using the methodological quality and synthesis of case series and case reports tool designed by Murad *et al.*, since all included studies were non-comparative single case reports or case series [7]. According to this instrument, each study is evaluated based on four domains: selection of study groups, ascertainment, causality, and reporting. We kept items related to the selection of cases, ascertainment, and reporting; and we removed three items from the causality domain (challenge/re-challenge phenomenon, dose–response effect, and long follow-up for outcomes to occur) because they are not relevant to this review (Supplementary File 2). This resulted in a five-item tool to assess whether the methodological quality of included studies is good, unclear, or low based on three possible answers for each item (yes, cannot tell, no). This tool has been previously applied with consistency among reviewers [8–10]. The same two reviewers assessed the methodological quality of included studies with discussion and adjudication by the corresponding author in case of disagreement.

## Results

### The frequency of pouch volvulus after IPAA

Three studies reporting the long-term outcome of at least 1,000 patients with IPAA were published [11–13]. Pouch volvulus was reported in 3 of 1,700 patients (0.18%) in one study [11]. Primary and corresponding authors of the remaining two studies were contacted and data were not available for reporting. Therefore, it was not possible to estimate the frequency from the two other studies [12, 13].

### Study characteristics

**Figure 1.**
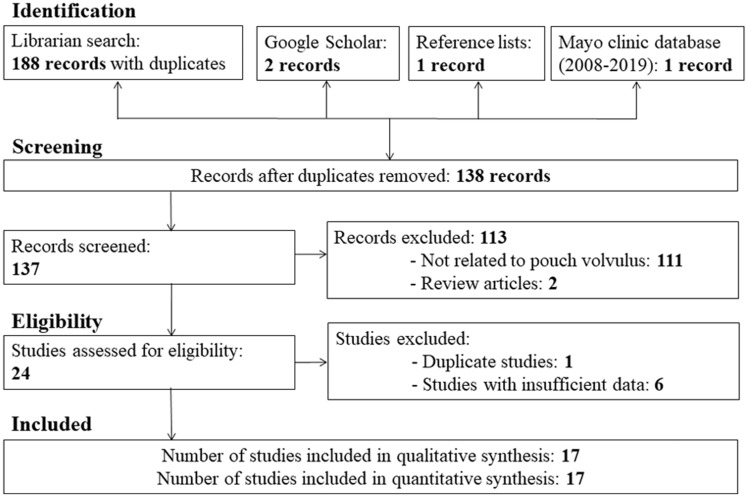
Flow diagram through the different phases of the systematic review.

The flow diagram of different phases of this systematic review is shown in Figure 1. We identified 16 publications from nine countries published between 1993 and 2018 that met the selection criteria [14–29] and we reported an additional case from the Mayo Clinic identified in the database search. Thirteen studies were found by the librarian search, two by the Google Scholar search [14, 19], and one from reference lists of relevant papers [28]. All the publications were in English. Two publications were in abstract form [22, 27], four were letters to the editor [14, 16, 23, 24], and the remaining were full-text articles. There was one case series reporting six patients [26] and 15 case reports reporting one patient each. We excluded one duplicate study [23] and six studies reporting 11 patients because of insufficient data about pouch volvulus [11, 30–34].

### Patient characteristics

Table 1 shows the main characteristics of the included patients. We identified 22 patients in total with a median age of 32 (range, 22–71) years. Sixteen patients were female (73%). Eight-one percent of patients were Caucasian and 13% were Asian. The indication of IPAA was reported in 21 patients: medically refractory UC (18 patients), FAP (2 patients), and rectal cancer (1 patient). J-pouch was constructed in 19 patients and W- and S-pouch in one patient each. The type of pouch was not reported in one patient [28], but the radiological and endoscopic findings suggested a J-pouch type. BMI at time of presentation was reported in six patients in the case series of Landisch *et al*. [26] with a median value of 19.8 (range, 17.5–24.0) kg/m^2^ and was not reported in the remaining patients.

**Table 1.** Main characteristics of the included patients

First author/year	Country	No.	Age/ gender	IPAA indication	Pouch type	IPAA volvulus, month	Treatment of volvulus	Follow-up, month	Volvulus recurrence
Poggioli/1993 [28]	Italy	1	23/female	NR	J	0.5	Upper pouch excision Redo IPAA	60	No
Swarnkar/2004 [14]	UK	1	34/female	UC	J	NR	Pouch defunction Perineal ileostomy	36	No
Ullah/2007 [15]	UK	1	22/male	UC	W	36	Pouch excision	NR	No
Jain/2009 [16]	USA	1	25/male	UC	J	20	Pouch excision Redo IPAA	2	No
Warren/2011 [17]	UK	1	39/female	UC	NR	120	Pouch-pexy	8	Yes
Choughari/2010 [18]	Belgium	1	35/female	FAP	J	156	Upper pouch excision Ileostomy	2	No
George/2014 [19]	USA	1	34/female	UC	J	36	Pouch excision Redo IPAA	2	No
Myrelid/2014 [29]	Sweden	1	58/female	UC	J	132	1st surgery: pouch-pexy 2nd surgery: pouch-pexy	10	Yes
Tyagi/2014 [20]	India	1	28/male	UC	S	NR	Pouch-pexy	6	No
Arima/2014 [21]	Japan	1	65/female	UC	J	180	Pouch-pexy	5	No
Abraham/2015 [22]	USA	1	70/male	UC	J	NR	Pouch-pexy	NR	No
Cárdenas/2016 [23]	Spain	1	36/female	FAP	J	108	1st surgery: pouch-pexy 2nd surgery: detorsion 3rd surgery: redo IPAA	4	Yes
Lee/2015 [24]	Hong Kong	1	71/male	RC	J	36	Pouch-pexy	6	Yes
Mullen/2016 [25]	USA	1	37/female	UC	J	120	Pouch detorsion	24	No
Landisch/2018 [26]	USA	6	Median, 31 5 females 1 male	UC	J	24	Endoscopic detorsion 1 Pouch detorsion 1 Pouch-pexy 1 Pouch excision 3 Redo IPAA 1	28	No
Ghouri/2018 [27]	USA	1	30/female	UC	J	2	Endoscopic incision	0.5	No
Mayo/2018	USA	1	32/female	UC	J	125	Pouch detorsion	5	No
Total 16 studies 1 Mayo Clinic patient	9 countries	22	Median, 32 16 females 6 males	UC 18 FAP 2 RC 1 NR 1	J 19 W 1 S 1 NR 1	Median, 36	Surgery 20 Pouch-pexy 8 Pouch excision 8 Redo IPAA 5	Median, 9	4 (18%)

NR, not reported; UC, ulcerative colitis; FAP, familial adenomatous polyposis; RC, rectal cancer; IPAA, ileal pouch–anal anastomosis.

### Symptoms and serological exams

**Figure 2.**
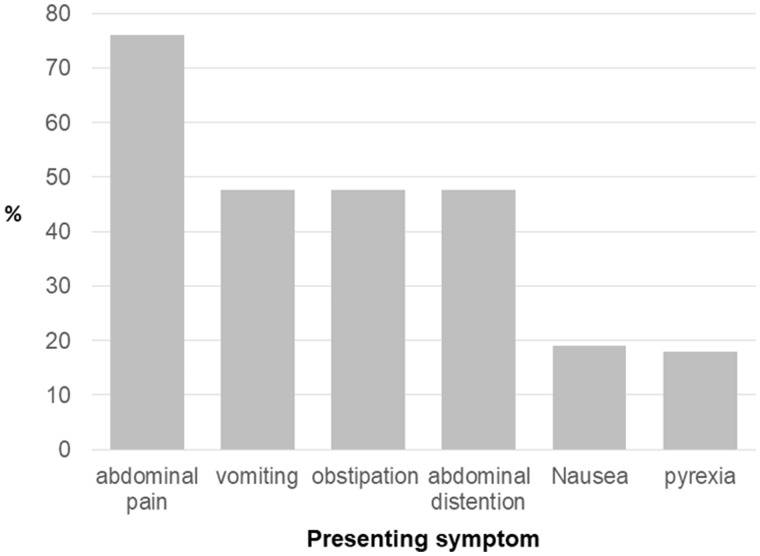
Distribution of index symptoms of patients with pouch volvulus

The median interval between the performance of IPAA and occurrence of pouch volvulus was 36 (range, 0.5–180) months. Abdominal pain was the most commonly reported symptom followed by obstipation, abdominal distention, and vomiting (Figure 2). Metabolic acidosis was reported in one patient who was diagnosed initially as pouchitis with a delay of 84 hours before surgical intervention. A gangrenous pouch was found at surgery and pouch excision with ileostomy was performed [15].

### Diagnosis of pouch volvulus

Table 2 shows the diagnostic procedures performed in included patients and their results. Abdominal CT, Gastrograffin enema, colonoscopy, and plain abdominal radiograph allowed the diagnosis to be made in 13 of 17 patients (76%), 11 of 17 patients (65%), 6 of 10 patients (60%), and 3 of 10 patients (30%), respectively. No signs of pouch ischemia or leak were noted on abdominal CT. Colonoscopy was performed after tube insertion and resolution of the volvulus in one patient. Pouchitis and/or pouch ulceration were noted in seven patients who underwent colonoscopy. Ischemia of the distal aspect of IPAA was observed in one patient on colonoscopy [16]. The volvulus was along the longitudinal axis of the pouch in 15 of 16 patients with available data (94%) and along its transverse axis in one patient (6%). The median interval between symptom onset and diagnosis of volvulus was 24 (range, 12–82) hours in 19 patients with available data.

**Table 2.** Diagnostic procedures and their results in included patients

Diagnostic procedures	No. of patients	Findings	Volvulus diagnosis, *n* (%)
Plain abdominal radiograph	10	Bird beak sign: gaseous pouch distention with a few scattered small-bowel loops	3 (30%)
Gastrografin enema	10	Bird beak sign: gradual narrowing/tapering of pouch up to the level of obstruction during contrast/barium insertion to the rectum corkscrew configuration: spiral appearance of the pouch (Figure 4B)	6 (60%)
Abdominal CT	17	Whirl sign: dilated intestine consistent with a distal bowel obstruction due to rotation of the pouch around its axis (Figure 4A)	13 (76%)
Colonoscopy	17	Spoke-wheel sign: a soft-tissue mass with radiating mucosal folds simulating a ‘spoke wheel’	11 (65%)

### Endoscopic treatment of pouch volvulus

Endoscopic treatment was applied in 12 patients. Endoscopic detorsion was performed in 11 patients and was successful in 7, partially successful in 2, and unsuccessful in 2 patients. In one patient, intra-operative endoscopic detorsion coupled with laparoscopic manipulation of the pouch allowed reduction of the volvulus [22]. When implemented as a sole modality of treatment, endoscopic detorsion was successful in one patient who refused surgical intervention and remained free of relapses during 3-year follow-up [26].

One patient received circumferential electro-incision/cauterization by endoscopic needle knife of the pouch to relieve a partial stenosis at the IPAA-anastomosis. In this patient, pouchoscopy was performed after 2* *weeks, showing resolution of the obstruction [27].

### Surgical treatment of pouch volvulus

Twenty patients were treated surgically. An open laparotomy was performed in 10 patients, laparoscopic surgery in 2, and the surgical approach was not reported in 8 patients. In three patients, detorsion of the pouch was performed and the mesenteric defect was closed, without concomitant pouch-pexy [25, 26] (Mayo Clinic patient). Pouch-pexy and pouch excision with ileostomy were performed in eight patients each. A redo IPAA was offered to five patients after pouch-pexy or pouch excision. In one case, the anastomosis was defunctioned with a perineal ileostomy [14] (Table 1).

The volvulus resulted in secondary complications in 5 of 22 patients (23%): mild ischemic pouch (*n *=* *1), ischemia and fistula of the pouch (*n *=* *1), gangrenous pouch with minor perforation and metabolic acidosis (*n *=* *1), pouch perforation (*n *=* *1), and small-bowel infarction with septic shock (*n *=* *1). Two post-operative complications were reported. One patient developed a mild anastomotic stricture treated by digital dilation and a second patient developed an anastomotic leak, pelvic sepsis, and entero-cutaneous fistula after pouch excision and redo IPAA [26].

### Follow-up and outcome

The median follow-up after endoscopic or surgical treatment was 9 (range, 0.5–60) months. Recurrence of pouch volvulus after successful endoscopic detorsion was observed in two of seven patients (28.5%). Recurrence of pouch volvulus was observed in 2 of 20 (10%) patients treated surgically. It occurred 2 and 10* *months after the first surgical intervention and was treated by repeated pouch-pexy in the first patient and by detorsion and redo IPAA in the second patient [23, 29]. No mortality was reported during the period of follow-up.

### Assessment of the methodological quality of the included studies

**Figure 3.**
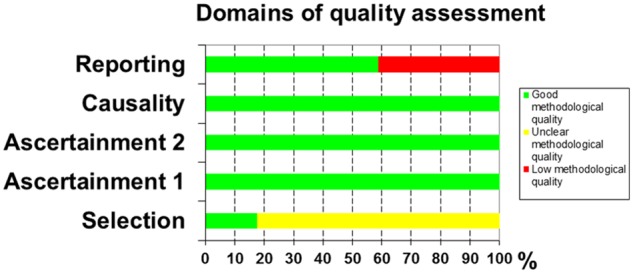
Evaluation of the methodological quality of the studies in the systematic review

Supplementary File 3 shows the assessment of the methodological quality of included studies and Figure 3 shows the overall evaluation of the methodological quality of these studies. For the selection domain, most authors did not mention whether the reported cases represented the whole experience of their centers. The agreement of the two reviewers in assessing the methodological quality of included studies was 92%.

### Generalizability of the results

The small sample size limits the generalizability of results of this review to all cases of pouch volvulus after IPAA. However, the rarity of this complication, the good-quality assessment of the majority of included studies, and the fact that the included patients belonged to different ethnicities could make these results applicable in the appropriate context.

## Discussion

Poggioli *et al*. [28] reported the first case of pouch volvulus in a patient with IPAA more than 25 years ago. Unlike post-surgical small-bowel obstruction, pouch volvulus is not typically attributed to post-operative adhesive disease, but rather the pouch remains free and mobile within the pelvis and torsion may occur as a result of a longer mesenteric root, mesenteric stretch above the retroperitoneal plane, or a defect in the mesentery [19, 21]. Although pouch volvulus is thought to be exceedingly rare [35], this systematic review identified 22 studies with a total of 33 patients in the published literature (Supplementary Figure 1). Fourteen of these studies were reported in the last 5* *years, which may reflect an increased awareness of this rare complication of IPAA. We include in this systematic review 16 studies published between 1993 and 2018 and a total of 21 cases of pouch volvulus with sufficient clinical data, adhering to rigorous a priori inclusion criteria, in addition to one patient from the Mayo Clinic, identified within 10 years.

Pouch twisting has also been described in the literature, at times distinctly differentiated from pouch volvulus, as suggested by Lipman *et al*. [6]. A pouch twist may be partly related to the anastomosis and how this was constructed at the time of surgery. Therefore, a twist may be created surgically. A volvulus, on the other hand, is pouch torsion with the orientation of the anastomosis in the appropriate configuration. Other experts, however, have considered both designations representative of one entity [36]. In our systematic review, we excluded cases of intra-operative twisted pouch, restricting our review to pouch volvulus. It is also important to distinguish pouch volvulus, which is, by definition, an acute torsion of the pouch around its axis, likely at the level of the pouch inlet, from afferent limb syndrome, which signifies angulation in the afferent limb, intermittently leading to chronic and variable degrees of partial small-bowel obstructions.

Pouch volvulus can occur following the creation of any pouch configuration, as demonstrated in this systematic review, although the majority occurred following J-pouch creation, which likely reflects the popularity of the J figure for IPAA [37]. Whether one configuration is more prone to volvulus than others is unclear [36].

It is noteworthy to mention that the majority of patients did not develop intestinal obstruction symptoms and rather presented with nonspecific manifestations such as abdominal pain or nausea. Obstructive features, such as abdominal distention, obstipation, or vomiting, occurred in fewer than half of patients. Moreover, laboratory tests were within normal limits at the time of emergent presentation in all reported cases. The totality of nonspecific symptoms, lack of laboratory abnormalities, in addition to the possibility of a normal digital rectal exam (the axis of rotation may not be reachable) may incur a serious delay in diagnosis, leading to catastrophic results, such as necrosis or ischemia, leading to pouch excision. This was reported in one case in this systematic review [15].

Several risk factors may contribute to the development of pouch volvulus and can be divided into patient-related factors, procedure-related factors, and combined factors. Patient-related factors include body habitus (e.g. low BMI) [26], pelvic anatomy, and development of pelvic-floor dysfunction after pouch surgery. Procedure-related factors entail those of pouch configuration (e.g. pouch volume, orientation, laparoscopic creation, fixation within the pelvic cavity, presence of a mesenteric defect) [23, 25, 36]. Combined factors relate to motility disturbance following the creation of IPAA construction and anastomosis creation between the anus (cuff transition zone) and the enteral pouch, as it may result in augmented contractions and enteral dysmotility, increasing the occurrence of volvulus [36, 38]. Other factors, such as defecation dyssynergia and pouch dysfunction, are likely in play. Such motility dysfunctions occur in 75% of IPAA patients, especially in patients with chronic pouchitis, and have been implicated as a putative mechanism in inducing it [39]. Pelvic dyssynergia is thus particularly paramount to identify and treat, since studies have suggested that chronic pouch ischemic changes may represent a prodromal phase for a future episode of volvulus [26].

**Figure 4.**
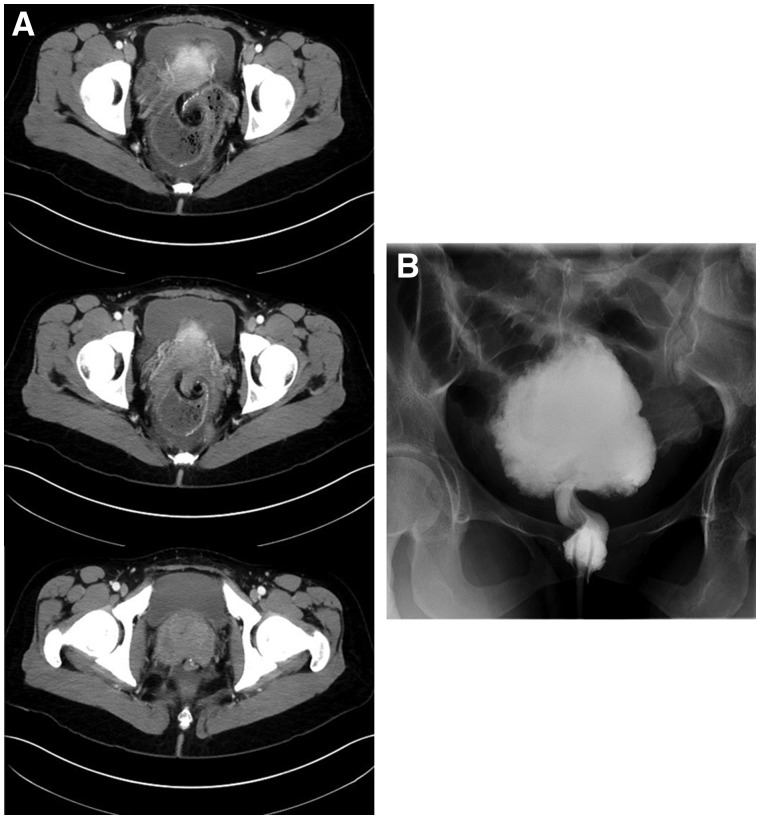
Imaging characteristics of pouch volvulus. (A) Computed tomography showing swirling of the distal pouch and its mesentery just above the ileoanal pouch anastomosis. (B) Water-soluble contrast enema showing a volvulus in the lower ileoanal pouch, with marked dilation of the proximal ileoanal pouch. On this exam, the volvulus could not be reduced with administration of Hypaque enema. The ileoanal anastomosis is patent and no leakage of contrast is observed.

**Figure 5.**
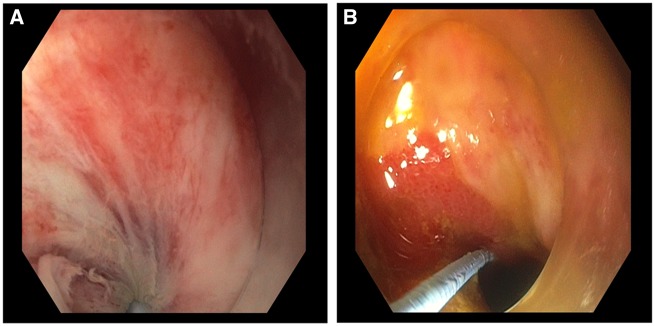
Endoscopic views during endoscopic attempt to reduce a pouch volvulus. (A) Swirling mucosa is seen converging towards the pinpoint torsed lumen. (B) Improvement in luminal torsion after endoscopic reduction.

In this review, the diagnosis of IPAA volvulus could not be based on clinical symptoms and appropriate endoscopic/imaging studies should be requested early. In this systematic review, computed tomography was the modality of choice for the diagnosis of pouch volvulus, demonstrating typical small-bowel torsion signs around the mesenteric axis [40] (Figure 4). Interestingly, magnetic resonance imaging was not pursued in any of the cases, likely reflecting the lack of immediate availability in the emergency-department context. Furthermore, bedside pelvic ultrasound was not performed in the reported patients. If clinical suspicion arises, flexible pouchscopy can be pursued, although endoscopic detorsion was not found to be generally successful, due to volvulus recurrence in the majority of reported cases. However, endoscopy may be critical to assess end-organ damage and viability of the anastomotic mucosa, with a high diagnostic yield [41] (Figure 5).

Surgical intervention was performed in 91% of patients. Although no patient mortality was reported, pouch excision was pursued in 36%. The management of the volvulus depends on the viability of the pouch, which could be determined initially via endoscopy. If the pouch is viable and a successful endoscopic reduction of the volvulus was performed, the placement of a decompression tube could optimize the outcome of definitive operative management [6]. Pouch detorsion and pexy may provide satisfactory surgical results. Excision of the pouch is necessary in case of necrosis. Recreation of an ileal pouch should be generally avoided in the acute setting, but it may be necessary in cases of recurrent pouch volvulus [6]. The identification and treatment of instigating events, such as reducing hernias or closing mesenteric defects, are crucial to avoid the volvulus recurrence.

This systematic review has several inherent shortcomings. First, it is based on case series and case reports with a very high likelihood of selection bias [7]. Second, missing clinical data and insufficient follow-up in some reports precluded gathering complete information. Third, the small sample size of included patients precludes the generalizability of our results to all cases of pouch volvulus. We attempted to compensate for some of these shortcomings by following a rigorous a priori protocol and by systematic examination of major databases, with an exhaustive search strategy that was not restricted to language or manuscript type. In the absence of higher evidence, evidence from case reports and case series becomes more significant [7]. We also attempted to synthesize the pooled information and present it in the context of a differential diagnosis framework to increase the clinical awareness of this rare, but serious, complication of IPAA surgery.

To the best of our knowledge, this is the first systematic review that synthesizes the evidence on pouch volvulus in patients with IPAA. This review followed an a priori protocol with evaluation of multiple databases by pairs of independent reviewers. Prospective longitudinal studies, examining the natural history following pouch surgery will be able to offer more accurate data for the frequency, risk factors, and therapeutic options for IPAA complications.

In conclusion, pouch volvulus is a rare but serious complication of IPAA. Heralding signs and symptoms, such as recurrent vague abdominal pain after IPAA surgery and acute obstipation, should initiate a prompt workup for a pouch volvulus. Quick and early application of barium enema, endoscopy, or cross-sectional imaging should be pursued. Endoscopic therapy often fails and salvage surgical pouch detorsion with pouch-pexy has a good outcome when performed early.

## Funding

No source of funding has been declared by the authors. The guidelines of the PRISMA 2009 statement were adopted.

## Conflicts of Interest

L.H.R.: Pfizer Pharmaceuticals, consultant, honorarium paid to Mayo. P.D.: Speaker's bureau: Abbvie; Advisory board: Pfizer and Janssen; Research grant: Takeda. A.H.L.: Takeda, consultant. All other authors have no conflict of interest or disclosures.

## Supplementary Material

goz045_Supplementary_Data
