# Supplementary material for: Volvulus of the ileal pouch–anal anastomosis: a meta-narrative systematic review of frequency, diagnosis, and treatment outcomes
Source: Gastroenterol Rep (Oxf). 2019 Sep 17;7(6):403–10. doi: 10.1093/gastro/goz045 (PMC6911998; doi:10.1093/gastro/goz045)
Supplement: goz045_Supplementary_Data [file goz045_supplementary_data.zip › goz045-Suppl_data/Supplementary_Data.docx]

**Supplementary Files**

**Supplementary File 1. Data sources and search strategies**

A comprehensive search of several databases from each database’s inception to May 14th, 2018, any language was conducted. The databases included Ovid MEDLINE Epub Ahead of Print, Ovid Medline In-Process & Other Non-Indexed Citations, Ovid MEDLINE, Ovid EMBASE, Ovid Cochrane Central Register of Controlled Trials, Ovid Cochrane Database of Systematic Reviews, and Scopus. The search strategy was designed and conducted by an experienced librarian with input from the study’s principle investigator. Controlled vocabulary supplemented with keywords was used to search for ileo-anal pouch volvulus. The actual strategy is available from the reprint author.

**Actual search strategies**

**Ovid**

Database(s): Embase 1988 to 2018 Week 20, EBM Reviews - Cochrane Central Register of

Controlled Trials April 2018, EBM Reviews - Cochrane Database of Systematic Reviews 2005 to May 9, 2018, Ovid MEDLINE(R) Epub Ahead of Print, In-Process & Other Non-Indexed

Citations, Ovid MEDLINE(R) Daily and Ovid MEDLINE(R) 1946 to Present.

Search strategy:

# Searches Results

1 exp Intestinal Volvulus/ 4369

2 (pseudovolvulus or volvulus or twist*).ti,ab,hw,kw. 64692

3 1 or 2 64692

4 exp Colonic Pouches/ 2002

5 ("ileal reservoir*" or "ileoanal reservoir*" or "ileo-anal reservoir*" or IPAA

or pouch*).ti,ab,hw,kw. 44215

6 4 or 5 44234

7 3 and 6 188

8 remove duplicates from 7 134

**Scopus**

1 TITLE-ABS-KEY(pseudovolvulus OR volvulus or twist*)

2 TITLE-ABS-KEY("ileal reservoir*" OR "ileoanal reservoir*" OR "ileo-anal reservoir*" OR IpAA OR pouch*)

3 1 and 2

4 INDEX(embase) OR INDEX(medline) OR PMID(0* OR 1* OR 2* OR 3* OR 4* OR 5* OR 6* OR 7* OR 8* OR 9*)

5 3 and not 4

**Supplementary File 2. Tool for assessment of methodological quality of case reports and case series (adapted from Murad *et al*. [1])**

| **Domains** | **Leading explanatory questions** |
| --- | --- |
| **Selection** | Does the patient(s) represent(s) the entire experience of the researchers or is the selection modality unclear to the extent that other patients with similar presentation may have been missed? |
| **Ascertainment** | 1. Was the exposure sufficiently ascertained?  2. Was the outcome sufficiently ascertained? |
| **Causality** | Were other plausible causes that may account for the observation ruled out beyond a reasonable doubt? |
| **Reporting** | Is the case(s) relayed with adequate details to allow other investigators to replicate the research or to permit practitioners to make inferences related to their practice? |

1. Murad MH, Sultan S, Haffar S, Bazerbachi F. Methodological quality and synthesis of case series and case reports. *BMJ Evid Based Med* 2018; **23**:60-63.

**Supplementary File 3. Assessment of methodological quality of included studies**

| **First author/year** | **No.** | **Selection** | **Ascertainment 1** | **Ascertainment 2** | **Causality** | **Reporting** |
| --- | --- | --- | --- | --- | --- | --- |
| **Poggioli/1993[1]** | 1 | 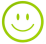 | 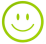 | 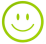 | 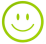 | 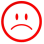 |
| **Swarnka/2004 [2]** | 1 | 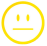 | 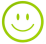 | 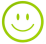 | 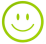 | 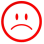 |
| **Ullah/2007 [3]** | 1 | 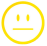 | 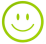 | 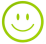 | 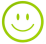 | 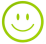 |
| **Jain/2009 [4]** | 1 | 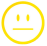 | 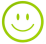 | 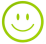 | 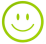 | 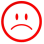 |
| **Warren/2011 [5]** | 1 | 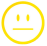 | 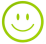 | 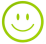 | 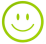 | 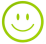 |
| **Choughari/2010 [6]** | 1 | 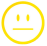 | 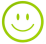 | 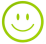 | 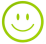 | 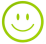 |
| **George /2014 [7]** | 1 | 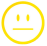 | 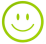 | 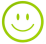 | 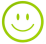 | 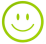 |
| **Myrelid/2014 [8]** | 1 | 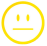 | 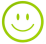 | 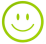 | 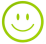 | 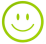 |
| **Tyagi/2014 [9]** | 1 | 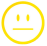 | 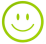 | 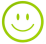 | 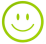 | 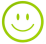 |
| **Arima/2014 [10]** | 1 | 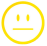 | 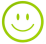 | 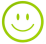 | 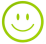 | 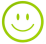 |
| **Abraham/2015 [11]** | 1 | 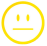 | 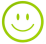 | 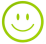 | 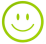 | 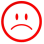 |
| **Cardenas/2015 [12]** | 1 | 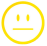 | 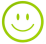 | 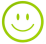 | 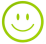 | 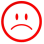 |
| **Lee/2015 [13]** | 1 | 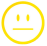 | 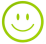 | 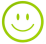 | 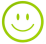 | 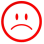 |
| **Mullen/2016 [14]** | 1 | 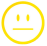 | 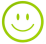 | 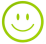 | 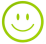 | 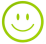 |
| **Landisch/2018 [15]** | 6 | 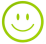 | 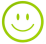 | 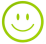 | 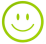 | 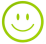 |
| **Ghouri/2018 [16]** | 1 | 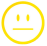 | 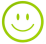 | 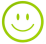 | 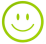 | 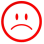 |
| **Mayo Clinic/2018** | 1 | 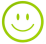 | 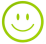 | 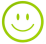 | 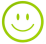 | 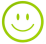 |
| **Total: 17 studies** | **22** | **GMQ 3**  **UMQ 14**  **LMQ 0** | **GMQ 17**  **UMQ 0**  **LMQ 0** | **GMQ 17**  **UMQ 0**  **LMQ 0** | **GMQ 17**  **UMQ 0**  **LMQ 0** | **GMQ 10**  **UMQ 0**  **LMQ 7** |

Good methodological quality – Unclear methodological quality – Low methodological quality.


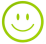

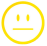

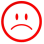


GMQ: good methodological quality – UMQ: unclear methodological quality – LMQ: low methodological quality.

1. Poggioli G, Marchetti F, Selleri S *et al.* Redo pouches: salvaging of failed ileal pouch-anal anastomoses. *Dis Colon Rectum* 1993;36:492-6.
2. Swarnkar K, Hopper N, Ryder J *et al*. 3 years follow-up of a twisted ileoanal pouch. *Colorectal Dis* 2004;6:133-4.
3. Ullah MZ, Fajobi OA, Bhargava AM. Long-axis rotational volvulus in a W ileoanal pouch: an unusual but potentially preventable problem. Report of a case. *Dis Colon Rectum* 2007;50:540-3.
4. Jain A, Abbas MA, Sekhon HK *et al*. Volvulus of an ileal J-pouch. *Inflamm Bowel Dis* 2010;16:3-4.
5. Warren C, O’Donnell ME, Gardiner KR *et al*. Successful management of ileo-anal pouch volvulus: Case reports. *Colorectal Dis* 2011;13:106-7.
6. Choughari L, Sohawon S, Noordally SO. Volvulus of an ileoanal J pouch. *Int J Colorectal Dis* 2010;25:1021-2.
7. George VV, Fajardo A. Long-axis rotational volvulus in an ileal J-pouch anal anastomosis: A preventable rare complication. *Case Rep Clin Med* 2014;3:28-31.
8. Myrelid P, Druvefors P, Andersson P. Recurrent Volvulus of an Ileal Pouch Requiring Repeat Pouchopexy: A Lesson Learnt. *Case Rep Surg* 2014;2014:1-3.
9. Tyagi G, Gupta U, Verma A *et al*. Volvulus of ileal S-pouch: A rare complication of ileal pouch anal anastomoses. *Int J Surg Case Rep* 2014;5:717-9.
10. Arima K, Watanabe M, Iwatsuki M *et al*. Volvulus of an ileal pouch-rectal anastomosis after subtotal colectomy for ulcerative colitis: report of a case. *Surg Today* 2014;44:2382-4.
11. Abraham G, Rider P. Endoscopic reduction of ileal J pouch volvulus with laparoscopic pexy. *Am J Gastroenterol* 2015;110:S693.
12. Cárdenas G, Bravo R, Delgado S *et al*. Recurrent volvular herniation of the ileal pouch: a case report and literature review. *Int J Colorectal Dis* 2016;31:749-50.
13. Lee SY, Fok KL, Cheung HYS *et al*. Laparoscopic rectopexy for recurrent volvulus of J pouch after total proctocolectomy and ileal pouch anal anastomosis: Recurrent volvulus of J pouch. *Surg Pract* 2015;19:133-6.
14. Mullen MG, Cullen JM, Michaels AD *et al*. Ileal J-Pouch Volvulus Following Total Proctocolectomy for Ulcerative Colitis. *J Gastrointest Surg* 2016;20:1072-3.
15. Landisch RM, Knechtges PM, Otterson MF *et al*. Pouch Volvulus in Patients Having Undergone Restorative Proctocolectomy for Ulcerative Colitis: A Case Series. Dis Colon Rectum 2018;61:713-8.
16. Ghouri YA, Shen B. P180 Endoscopic Correction of Twisted Pouch Related Stenosis. *Gastroenterology* 2018;154:S98.
