# Supplementary figures and images for: Volvulus of the ileal pouch–anal anastomosis: a meta-narrative systematic review of frequency, diagnosis, and treatment outcomes
Source: Gastroenterol Rep (Oxf). 2019 Sep 17;7(6):403–10. doi: 10.1093/gastro/goz045 (PMC6911998; doi:10.1093/gastro/goz045)

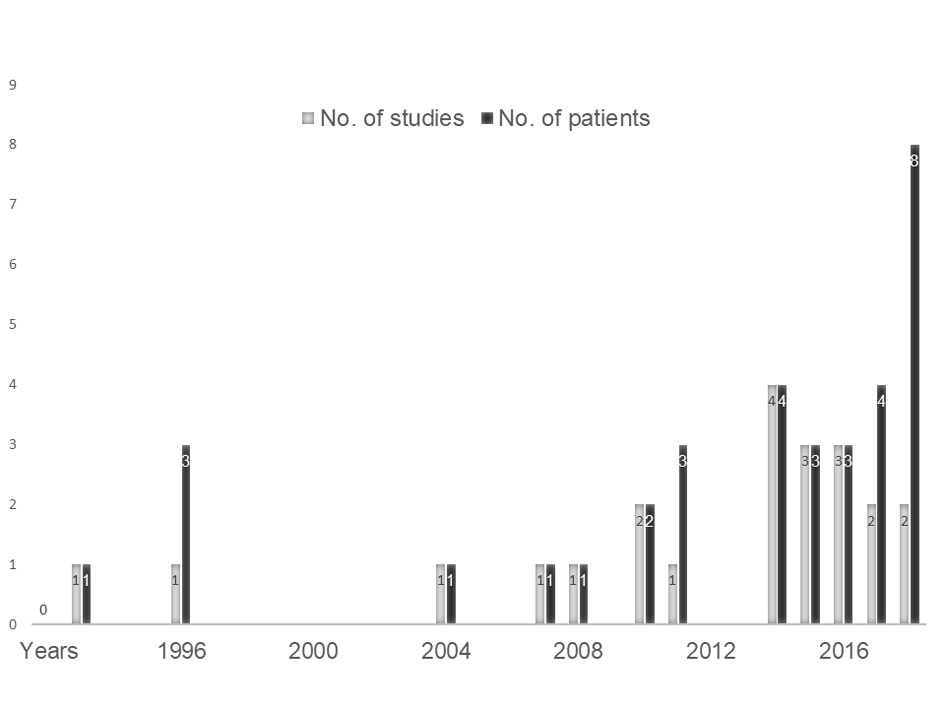

Supplement: goz045_Supplementary_Data [file goz045_supplementary_data.zip › goz045-Suppl_data/Supplementary_Figure.tif]
